# Long noncoding RNA NONHSAT122636.2 attenuates myocardial inflammation and apoptosis in myocarditis

**DOI:** 10.1371/journal.pone.0307779

**Published:** 2024-08-16

**Authors:** Yongjiao Liu, Li Zhang, Hailin Jia, Xinxin Feng, Mengjie Ma, Jing Wang, Bo Han

**Affiliations:** 1 Department of Pediatrics, Shandong Provincial Hospital, Shandong University, Jinan, Shandong, China; 2 Department of Pediatrics, Binzhou Medical University Hospital, Binzhou, Shandong, China; 3 Department of Pediatrics, Shandong Provincial Hospital Affiliated to Shandong First Medical University, Jinan, Shandong, China; Fujita Health University, JAPAN

## Abstract

**Objective:**

The main pathological change of myocarditis is an inflammatory injury of cardiomyocytes. Long noncoding RNAs (lncRNAs) are closely related to inflammation, and our previous study showed that differential expression of lncRNAs is associated with myocarditis. This study aimed to investigate the impact of lncRNAs on the onset of myocarditis.

**Methods:**

RNA expression was measured by quantitative reverse-transcription polymerase chain reaction (RT-qPCR). Lipopolysaccharide (LPS) was used to induce inflammation in human cardiomyocytes (HCMs). The expression of inflammatory cytokines and myocardial injury markers was detected by enzyme-linked immunosorbent assay (ELISA) and RT-qPCR. Cell viability and apoptosis were measured by the cell counting kit-8 assay and flow cytometry. The binding force between lncRNA NONHSAT122636.2 and microRNA miRNA-2110 was detected using the dual-luciferase assay.

**Results:**

NONHSAT122636.2 was dynamically expressed in patients with myocarditis and negatively correlated with inflammation severity. The overexpression of NONHSAT122636.2 improved inflammatory injury in LPS-stimulated HCMs. The study observed that there was a weak binding force between NONHSAT122636.2 and miR-2110.

**Conclusion:**

NONHSAT122636.2 attenuates myocardial inflammation and apoptosis in myocarditis. Additionally, its expression decreases in the peripheral blood of children suffering from myocarditis and in patients who are diagnosed for the first time showing higher diagnostic sensitivity and specificity. This decrease is negatively correlated with the degree of inflammation. Overall, the study suggests that NONHSAT122636.2 can be exploited as a potential diagnostic biomarker for pediatric myocarditis.

## Introduction

Myocarditis is characterized by inflammatory lesions in the heart muscle and can be induced by a variety of pathogenic infections [1, 2]. Early symptoms of myocarditis may not be prominently distinguishable, but immune cell sequestration, edema, necrotic cell death, and fibrosis are frequently detected around the lesion sites [7]. The clinical manifestations of myocarditis vary widely, and the disease can progress rapidly in some patients. Cardiogenic shocks and even sudden death may occur if myocarditis is not timely diagnosed and treated [3]. Notably, critically affected patients might progress to chronic dilated cardiomyopathy even after their apparent recovery [4, 5], which could negatively affect their quality of life in the long term. Therefore, early diagnosis and therapeutic intervention are crucial to prevent these patients from getting into further cardiac complications. Current diagnostic methods for myocarditis have limitations [2, 7]. Serum biomarker screening is first performed in suspected patients, but these biomarkers are mainly used for assessing prognosis and death risk due to their low sensitivity and specificity [6]. In myocarditis, electrocardiogram (ECG) and echocardiogram (Echo) cannot detect specific pathological lesions in the heart, which can be identified with cardiac magnetic resonance imaging (MRI) with high sensitivity [7]; however, MRI cannot effectively determine the etiology. Endomyocardial biopsy (EMB) is the gold standard for diagnosing myocarditis, but its application is limited by its invasive nature. Therefore, specific non-invasive diagnostic methods and therapies are urgently needed.

Noncoding RNAs (ncRNAs) were previously regarded as transcriptional garbage [8], and they refer to RNAs that are not translated into full-length functional proteins [9]. Recent studies have uncovered their functional and mechanistic importance in various pathomechanisms [10–13]. Long ncRNAs (lncRNAs) are longer than 200 nucleotides [14] and participate in pathophysiological processes including inflammation signaling [15], immune regulation [16], cell proliferation [17], and apoptosis [18]. LncRNAs are associated with the pathogenesis of cardiac dysfunctions such as myocardial infarction, heart failure and arrhythmias [19]. Liu C et al. reported that the lncRNA PVT1 induced hypoxic stress in cardiomyocytes by inhibiting the maturation of the microRNA (miRNA) miR-214-3p [20]. Han Y et al. demonstrated the role of lncRNA RMRP in preventing mitochondrial dysfunction and apoptotic death of cardiomyocytes via modulating the miR-1-5p/Hsp70 axis in a mouse model of lipopolysaccharide (LPS)-induced septic shock [21]. However, only a few studies have investigated the pathogenic roles of lncRNAs in the onset and progression of myocarditis.

We previously detected several differentially regulated lncRNAs in the peripheral blood of patients with myocarditis by microarray analysis [22]. These lncRNAs are the key regulators of disease-associated inflammatory pathways, including T-cell activation, mitogen-activated protein kinase (MAPK), and Janus kinase-signal transducers and activators of transcription (JAK-STAT) pathways. Based on the specific roles of these lncRNAs in the functional regulation of myocarditis-linked mRNA expression, we selected three differentially expressed lncRNAs, NONHSAT254241.1, NONHSAT242632.1, and NONHSAT122636.2, for further investigation. As shown in Table 1, all these lncRNAs were predicted to function through competitive endogenous RNAs (ceRNAs).

**Table 1:** Prediction information of three lncRNAs in microarray analysis.

lncRNA	Fold change (lnc)	*p* value	Subcellular location	miRNA	mRNA	Pathway
NONHSAT254241.1	0.46	0.04	Cytoplasm 70%	miRNA-3653-5p	CDK4	T cell receptor
NONHSAT243632.1	5.32	0.03	Cytoplasm 80%	miRNA-6749-3p	IL10RB	Jak-STAT
NONHSAT122636.2	0.35	<0.01	Cytoplasm 60%	miRNA-2110	CACNA1C	MAPK

## Materials and methods

### Patients and sampling

Peripheral venous blood samples were collected from 26 pediatric patients diagnosed with myocarditis and 26 age-matched healthy volunteers at the Pediatric Cardiology Department of Shandong Provincial Hospital (SPH) between October 2018 and December 2020, before the beginning of treatment. After collection, the red blood cells were lysed with a red blood cell lysate, followed by centrifugation to obtain leukocytes, which were then used for RNA extraction. Myocarditis cases were clinically diagnosed according to the recommendations of the European Society of Cardiology (ESC) working group on myocardial and pericardial diseases. Patients with other types of cardiovascular disorders, including coronary/congenital heart diseases, autoimmune-activation-related myocarditis, dilated/hypertrophic cardiomyopathy, and those who had been pre-treated with glucocorticoid or immune-suppressants, were excluded from the study. The clinical characteristics of the subjects are shown in Table 2. The study protocols were approved by the SPH Ethics Committee, and written informed consent was signed by the legal guardians/parents of all eligible child patients. The study did not involve the collection, use, or access to personally identifiable information.

**Table 2:** Clinical characteristics of the myocarditis and control groups.

	Myocarditis group	Control group	*p* value
Sex (male/female)	20/6	18/8	0.53
Age (years)	8.54±1.99	9.62±2.35	0.08
HS-TnT (pg/ml)	1563±1208	5.04±2.79	****
NT-proBNP (pg/ml)	7971±9646	74.35±34.14	***
CKMB-mass (ng/ml)	50.92±44.92	1.39±0.90	****
LVEF (%)	41.31±15.19	64.23±2.41	****

Hs-TnT, hypersensitive troponin T (normal range, 0–14 pg/ml); CKMB, creatine kinase muscle-brain isoenzyme (normal range 0.1–4.94 pg/ml); NT-pro BNP, NT-pro brain natriuretic peptide (normal range, 0–450 pg/ml); LVEF, left ventricular ejection fraction (normal value >60%).

***p< 0.001 versus control;

****p< 0.0001 versus control.

### Ethics statement

The authors state that they have obtained appropriate institutional review board approval from Shandong Provincial Hospital, Shandong University (No.2018-115), and have followed the principles outlined in the Declaration of Helsinki for all human or animal experimental investigations. We also confirm that informed consent has been obtained from all participants (or their legal guardians) involved in publishing this paper.

### Cell culture and LPS treatment

Human cardiomyocytes (HCMs) (Fenghui Biotech., China) were cultured in complete DMEM medium (Invitrogen, USA) supplemented with 1% penicillin-streptomycin (HyClone, USA) and 10% fetal bovine serum (FBS) (Gibco, USA) at 37°C and 5% CO_2_ under humidified conditions. HCMs were exposed to LPS to induce inflammation, simulate myocardial injury, and assess the expression of pathogenic markers, cell viability and apoptosis. To explore the differential expressions of lncRNAs in HCMs, we examined the effects of different concentrations of LPS over varying durations.

### RNA-fluorescent in situ hybridization (RNA-FISH) assay

The RNA-FISH assay (GenePharma, China) was conducted to determine subcellular localizations of target lncRNAs in HCMs. Briefly, cells were inoculated on coverslips in a 48-well plate at a density of 1×10^4^ cells/well and incubated for 24 hours, they were then fixed, washed and incubated with a probe overnight at 37°C. Subsequently, DAPI was added for nuclear staining and the fluorescence signals were captured by an OLYMPUS BX63 fluorescence microscope. The synthesized probe sequence (5’-T+TGGTAGAGGCGTGGCT+TTGAGGAC-3’) for NONHSAT122636.2 was purchased from GenePharma.

### RNA extraction and quantitative reverse-transcription polymerase chain reaction (RT-qPCR) assay

Total RNA from the peripheral blood leukocytes and cultured cells was extracted using SparkZol Reagent (SparkJade, Shandong, China). SparkZol was added to peripheral blood leukocytes and cultured cells, then the RNAs were extracted by chloroform, precipitated with isopropyl alcohol, washed with ethanol, and dissolved in water. RNA concentrations were determined using a NanoDrop ND-2000 spectrophotometer (NanoDrop, USA). cDNA was synthesized using the Evo M-MLV RT Premix for qPCR (Accurate Biotech, Hunan, China) and Hairpin-itTM miRNA and U6 snRNA Normalization RT-PCR Quantitation Kit (GenePharma, Shanghai, China). RT-qPCR was performed with SYBR Green Premix Pro Taq HS qPCR Kit (Accurate Biotech, Hunan, China) according to the manufacturer’s instructions on a LightCycler-480 system (Roche, Switzerland). We quantified the expression levels of three lncRNAs (NONHSAT254241.1, NONHSAT242632.1, and NONHSAT122636.2), as well as calcium voltage-gated channel subunit alpha1 C (CACNA1C), cyclin-dependent kinase 4 (CDK4), miRNA-2110, inflammatory cytokines such as interleukin 1β (IL-1β), interleukin 6 (IL-6), tumor necrosis factor-alpha (TNF-α), and myocardial injury markers such as creatine kinase MB (CKMB), cardiac troponin T (cTNT), and B-type natriuretic peptide (BNP), Expression levels were normalized against β-actin and RNU6B (U6) for lncRNAs/mRNAs and miRNAs, respectively, using the 2^-ΔΔCt^ method. Primers were purchased from BioSune (China) as shown in Table 3.

**Table 3:** Primer pairs for the qRT-PCR analysis.

Primer name	Forward primer (5’-3’)	Reverse primer (3’-5’)
NONHSAT254241.1	TTTGCCAACAGATCACCTTCT	AGCCCAGTTCACAGTCACAAC
NONHSAT243632.1	TCTTGGGCTAGATGGGAATGG	AGATGACAATGAGCCAGGAGG
NONHSAT122636.2	GTCAAGACACCCAGAGCACAA	GTAGACTTTCTCACATTCACCC
CDK4	TGGAAACTCTGAAGCCGACC	AAAGTCAGCATTTCCAGCAGC
CACNA1C	CCAAGACACGGCAAACAAGG	CGGTTGAAGAGGGACACGAA
miRNA-2110	GAGTGTAGTTGGGGAAACGGC	GTGCAGGGTCCGAGGT
IL-1β	GGATATGGAGCAACAAGTGGTGT	TTTCAACACGCAGGACAGGGTA
IL-6	CAATGAGGAGACTTGCCTGGT	GCAGGAACTGGATCAGGACT
TNF-α	CACTTTGGAGTGATCGGCCC	AGCTTGAGGGTTTGCTACAAC
CKMB	GCCATTCGGTAACACCCACA	TGTTTGCTGAGGTCGGGGTA
cTnT	GCGCTGGAAATAGAGCCTGG	TCCCCCATTTCCAAACAGGAGC
BNP	GCTTTCCTGGGAGGTCGTTC	TGGTTGCGCTGCTCCTGTAA
β-Actin	CATGTACGTTGCTATCCAGGC	CTCCTTAATGTCACGCACGAT
U6	CAGCACATATACTAAAATTGGAACG	ACGAATTTGCGTGTCATCC

CDK4, cyclin-dependent kinase 4; CACNA1C, calcium voltage-gated channel subunit alpha1 C; IL-1β, interleukin 1 beta; IL-6, interleukin 6; TNF- α, tumor necrosis factor alpha; CKMB, creatine kinase MB; cTNT, cardiac troponin T; BNP, B-type natriuretic peptide; and U6, RNU6B.

### Lentiviral transfections

A lentiviral vector carrying a GFP reporter and puromycin selection maker was used to overexpress NONHSAT122636.2 (LV-NONHSAT122636.2) (GeneChem, China) with a corresponding negative control vector (LV-NC) obtained for reference. Hitrans GP was used to enhance the viral infection capacity. The multiplicity of infection (MOI) value was optimized for downstream subsequent experiments. HCMs were cultured overnight in 24-well plates at a density of approximately 10000 cells/well. Viral transduction was performed with an MOI of 20, along with the enhancer. After 24 h of infection, the culture medium was harvested at 24 h intervals, up to 72 h. Infection efficiency was assessed using an ImageXpress confocal system and the expression levels were determined by qRT-PCR. Cells were selected using 5 μg/mL puromycin to stabilize lncRNA overexpression, with the death rate of LV-NC infected cells used as the reference. Stable strains were subsequently used for further experiments.

### Enzyme-linked immunosorbent assay (ELISA)

An ELISA kit (Elabscience, China) was used to measure the levels of inflammatory cytokines and myocardial injury markers in the supernatants of HCMs from all treatment and control groups. The optical density (OD) values were measured using a microplate reader (Bio-Tek Instruments, USA), and then converted into protein concentrations.

### Cell counting kit-8 assay

The cell counting kit-8 (CCK-8) (Dojindo, USA) was used to measure cell viability. Briefly, cells were seeded in 96-well plates at a density of 2000 cells/well, cultured for 24 h and then incubated for 2 h at 37°C after adding CCK-8 solution to each well. Absorbance was read at 450 nm on a Multiskan plate reader (Thermo-Fisher, USA) to determine cell viability.

### Flow cytometry

HCMs were treated with annexin V-phycoerythrin (PE)/7-amino actinomycin D (7AAD) reagents (BD Biosciences, USA), according to the manufacturer’s instructions. Apoptosis was analyzed using an FSCAN flow cytometer (BD Biosciences).

### Dual-luciferase reporter assay

Luciferase reporter vectors (Genechem, China) containing the wild-type (WT) or mutant (Mut) sequences of NONHSAT122636.2 (NONHSAT122636.2-WT and NONHSAT122636.2-Mut, respectively) were co-transfected with miR-2110 mimic or its negative control. Dual-luciferase activity was measured using the E1910 kit (Promega, USA) 48 hours post-transfection according to the kit manual. Results were normalized to Renilla luciferase activity.

### Statistical analyses

All experimental results were derived from three biological replicates, and data were presented as the mean ± standard deviation (SD). Comparison between the two groups was performed using a two-sided t-test using GraphPad Prism. A *p*-value of <0.05 was considered statistically significant.

## Results

### Identification of targeted lncRNA involved in myocarditis

**Fig 1:**
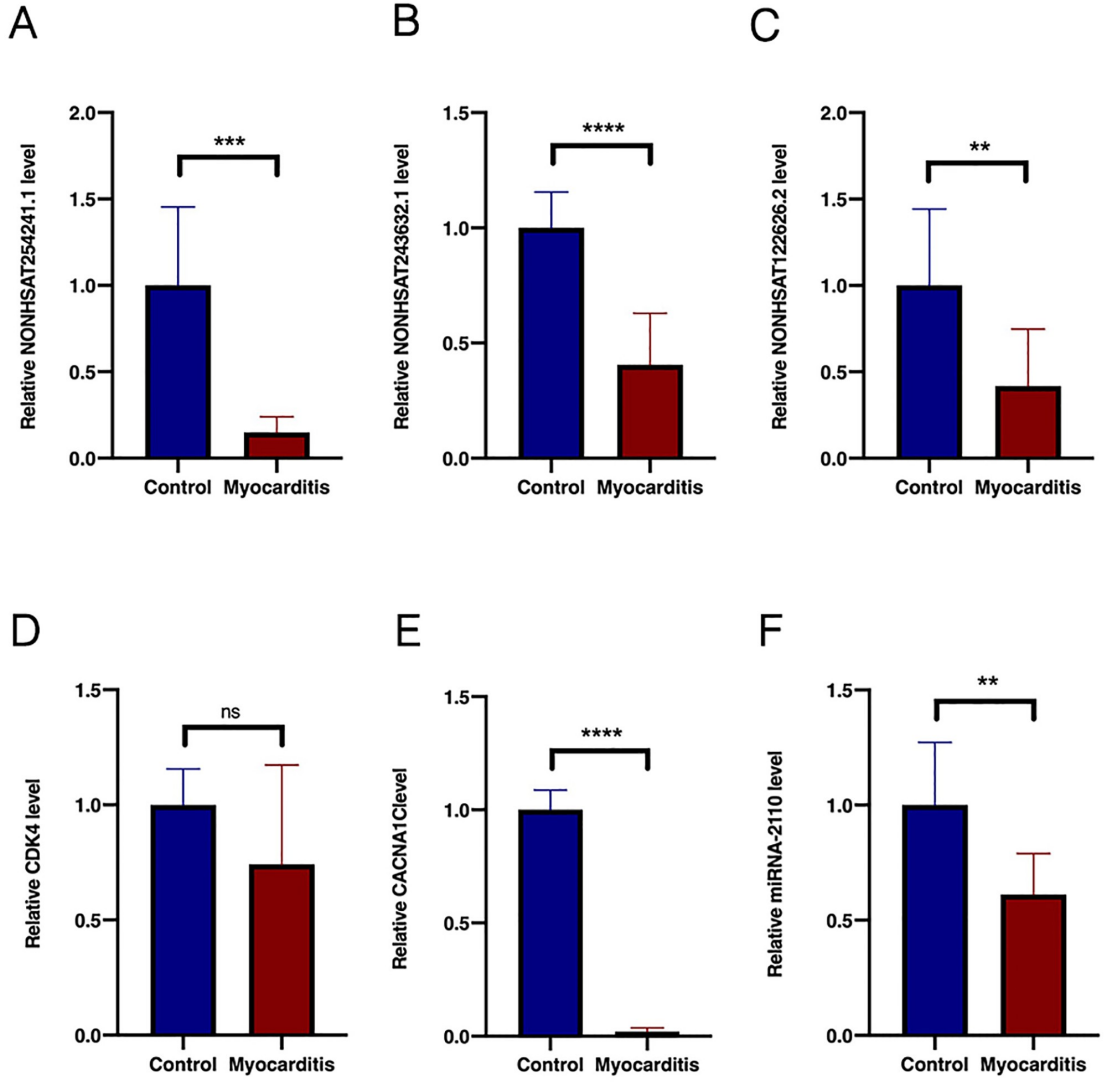
Validation of the relative expression levels of three lncRNAs, miR-2110, and CACNA1C by qRT-PCR. The expression levels of NONHSAT254241.1 (**A**), NONHSAT243632.1 (**B**), and NONHSAT122636.2 (**C**) were downregulated in samples from patients with myocarditis compared with those from controls. There was no significant difference in CDK4 expression between the myocarditis and control groups (**D**). The expression levels of CACNA1C (**E**) and miR-2110 (**F**) were downregulated in samples from patients with myocarditis compared with those from controls. Data met normal distribution and were presented as mean±SD. **p< 0.01 versus control; ***p< 0.001 versus control; ****p< 0.0001 versus control; ns (no significance), *p* > 0.05 versus control (Student’s t-test). lncRNA, long noncoding RNA, CDK4, cyclin-dependent kinase 4; CACNA1C, calcium voltage-gated channel subunit alpha1 C.

We collected blood samples from eight children with myocarditis and eight age-matched healthy children and measured lncRNAs by qRT-PCR analysis. Fig 1A & 1C showed that NONHSAT254241.1 and NONHSAT122636.2 were significantly downregulated in pediatric patients with myocarditis compared to healthy subjects. Fig 1B indicates that NONHSAT243632.1 expression was also reduced in patients with myocarditis contrary to our predicted result. *CDK4* and *CACNA1C* mRNA levels were lower in patients with myocarditis than in healthy controls (Fig 1D and 1E), consistent with our predicted result. However, the difference in CDK4 expression was not statistically significant. Finally, NONHSAT122636.2, miR-2110, and CACNA1C were further investigated.

### Expression pattern and predicted function of NONHSAT122636.2

**Fig 2:**
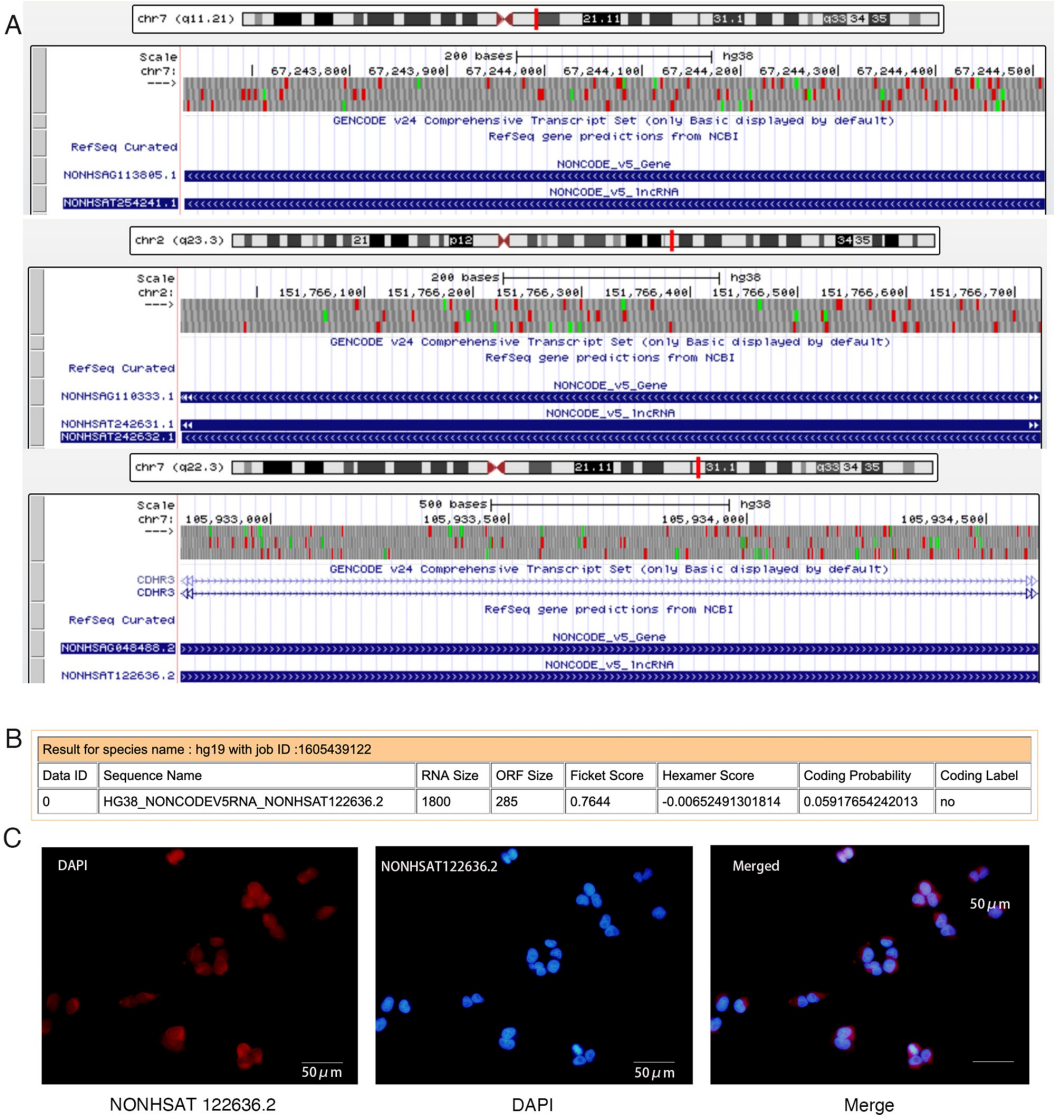
Characterization of the 3 lncRNAs. (**A**) Locations of NONHSAT254241.1, NONHSAT242632.1 and NONHSAT122636.2 on human chromosomes. (**B**) NONHSAT122636.2 was predicted to be a non-coding RNA by the CPAT program. (**C**) FISH assay revealed the predominant localization of NONHSAT122636.2 in the cytoplasm. NONHSAT122636.2 probes were labeled with Cy3 (red). Nuclei were stained with DAPI (blue). Scale bars, 50 μm. CPAT, the Coding-Potential Assessment Tool; FISH, fluorescence in situ hybridization; Cy3, cyanine 3.

As shown in Fig 2A, the lncRNA NONHSAT122636.2 is located on chromosome 7q22.3 (chr7:105932811–105934611). The Coding-Potential Assessment Tool (CPAT) indicated the absence of any open reading frames (ORFs) longer than 300 bp (Fig 2B). According to lncLocator database, subcellular localizations of NONHSAT122636.2 were primarily predicted to be cytoplasmic with a small amount in the nucleus, which was further confirmed by RNA-FISH assay (Fig 2C). Expression of NONHSAT122636.2 varied significantly between disease and control samples. It is predicted that this lncRNA may participate in MAPK signaling through the NONHSAT122636.2-miR-2110-CACNA1C axis.

### Reduced expression of NONHSAT122636.2 in children with myocarditis

**Fig 3:**
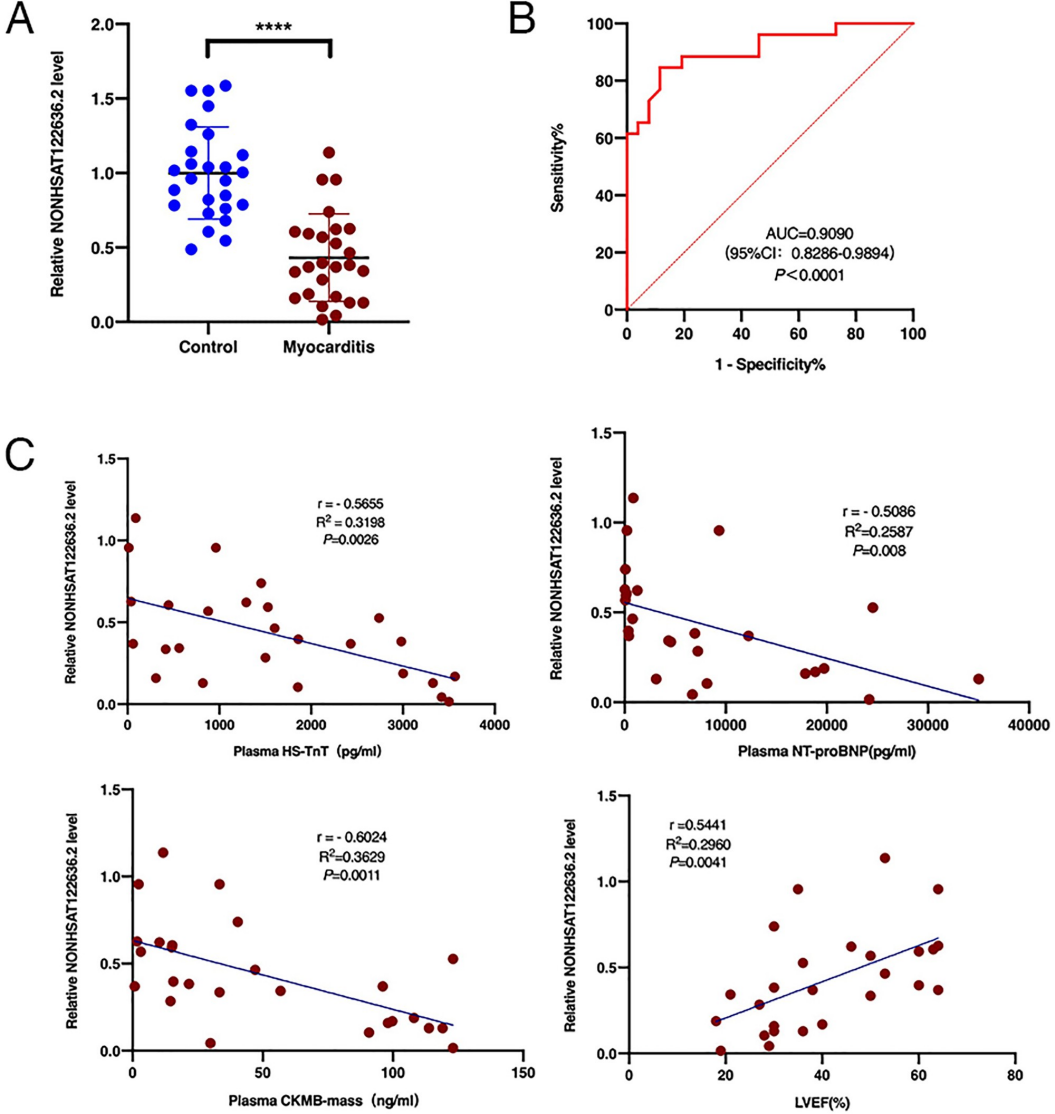
NONHSAT122636.2 is downregulated in patients with myocarditis. (**A**) The expression level of NONHSAT122636.2 was significantly downregulated in patients with myocarditis (n = 26) compared with controls (n = 26). (**B**) The ROC curve for NONHSAT122636.2 in patients with myocarditis. (**C**) The relative expression level of NONHSAT122636.2 was negatively correlated with the concentration of HS-TnT, NT-proBNP, and CKMB-mass in plasma, and it positively correlated with LVEF. Data met normal distribution and were presented as mean±SD. ****p< 0.0001 versus controls. AUC, area under the curve; R(r), Pearson correlation coefficient; ROC, receiver operating characteristic; creatine kinase MB (CKMB), cardiac troponin T (cTNT), and B-type natriuretic peptide (BNP), LVEF, left ventricular ejection fraction.

We collected peripheral blood samples from children with myocarditis and healthy children. Consistent with the microarray analysis, NONHSAT122636.2 expression significantly decreased in the peripheral blood of patients with myocarditis (n = 26) compared to healthy children (n = 26) (Fig 3A). Furthermore, the receiver operating characteristic (ROC) analysis indicated an area under the curve (AUC) of 0.9090 (cut-off value <0.65, p<0.0001, Fig 3B), suggesting that NONHSAT122636.2 could serve as a potential diagnostic biomarker for pediatric myocarditis.

### NONHSAT122636.2 correlated with the severity of myocarditis in children

To investigate the correlation of NONHSAT122636.2 expression and the severity of myocarditis, we collected clinical data, including HS-cTnT, NT-proBNP, CKMB-mass, and LVEF, from children with myocarditis and performed linear correlation analyses. Fig 3C shows that NONHSAT122636.2 expression was negatively correlated with serum levels of HS-cTnT, NT-proBNP, and CKMB-mass but positively correlated with LVEF in patients with myocarditis. These results suggest a negative correlation of NONHSAT122636.2 expression and the severity of myocarditis.

### Establishment of a myocardial injury model in HCMs by LPS treatment

**Fig 4:**
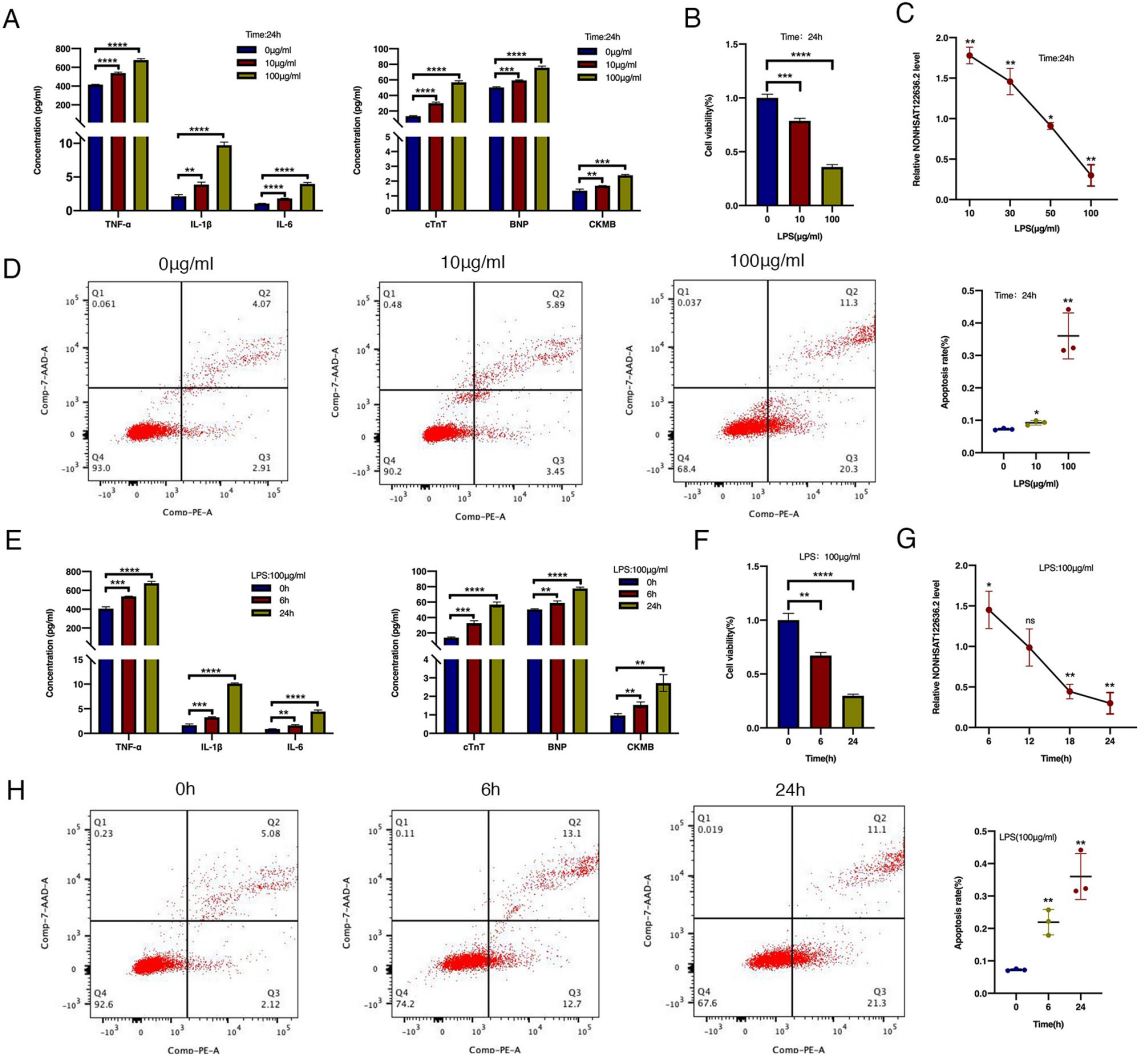
Expression of NONHSAT122636.2 in the myocardial injury model of LPS-stimulated HCMs. (**A)** Concentrations of inflammatory factors and myocardial injury markers after LPS stimulation for 24 h in HCMs. (**B, D**) Changes in the cell viability and apoptosis after HCMs were stimulated by LPS at different concentrations for 24 h. (**C**) Expression levels of NONHSAT122636.2 at different LPS concentrations (0, 30, 50,100 μg/mL) for 24 h. (**E**) Concentrations of inflammatory factors and markers of myocardial injury after HCMs were stimulated by 100 μg/mL LPS at different times. (**F, H**) Cell viability (CCK-8) and apoptosis (flow cytometry) were detected after HCMs were stimulated by 100 μg/mL LPS at different times. (**G**) Expression levels of NONHSAT122636.2 after 100 μg/mL of LPS exposure at different times (0, 12, 18, and 24 h). Data were presented as mean±SD. *p< 0.05 versus controls; **p< 0.01 versus controls; ***p< 0.001 versus controls; ****p< 0.0001 versus controls; ns (no significance), *p*>0.05 versus controls (Student’s t-test).

To further verify the function of NONHSAT122636.2 *in vitro*, we established a myocarditis model of cultured HCMs by LPS stimulation. HCMs were subjected to serum starvation for 12 h before being stimulated with 10 μg/mL of LPS for 24 h, and relevant markers were measured by qRT-PCR and ELISA methods. Compared to the control group, expression levels of inflammatory cytokines and myocardial injury markers were markedly upregulated after LPS stimulation (Fig 4A); cell viability was decreased (Fig 4B) and apoptosis was significantly increased (Fig 4D), indicating successful establishment of the myocardial injury model.

### NONHSAT122636.2 correlated with the severity of inflammation in LPS-treated HCMs

The cardiomyocyte inflammation model successfully mimicked the inflammatory phenotype of myocardial injury observed in patients. Increasing the concentration of LPS upregulated the expression of the inflammatory cytokines and myocardial injury markers (Fig 4A), decreased cellular viability further (Fig 4B), and increased apoptosis increased (Fig 4D), compared to the control group. Similarly, prolonging the treatment duration at a specific LPS concentration enhanced inflammatory and myocardial injury phenotypes in HCMs (Fig 4E, 4F & 4H). NONHSAT122636.2 expression decreased with the exacerbated inflammation and myocardial injury, both in response to increasing LPS-dose and prolonged treatment duration in HCMs compared to mock-treated cells (Fig 4C & 4G). Thus, NONHSAT122636.2 expression negatively correlated with the severity of inflammation in LPS-treated HCMs. Specifically, NONHSAT122636.2 expression was significantly decreased when HCMs were exposed to 100 μg/mL of LPS for 24 h.

### Overexpression of NONHSAT122636.2 attenuates LPS-induced cell inflammation and apoptosis in HCMs

**Fig 5:**
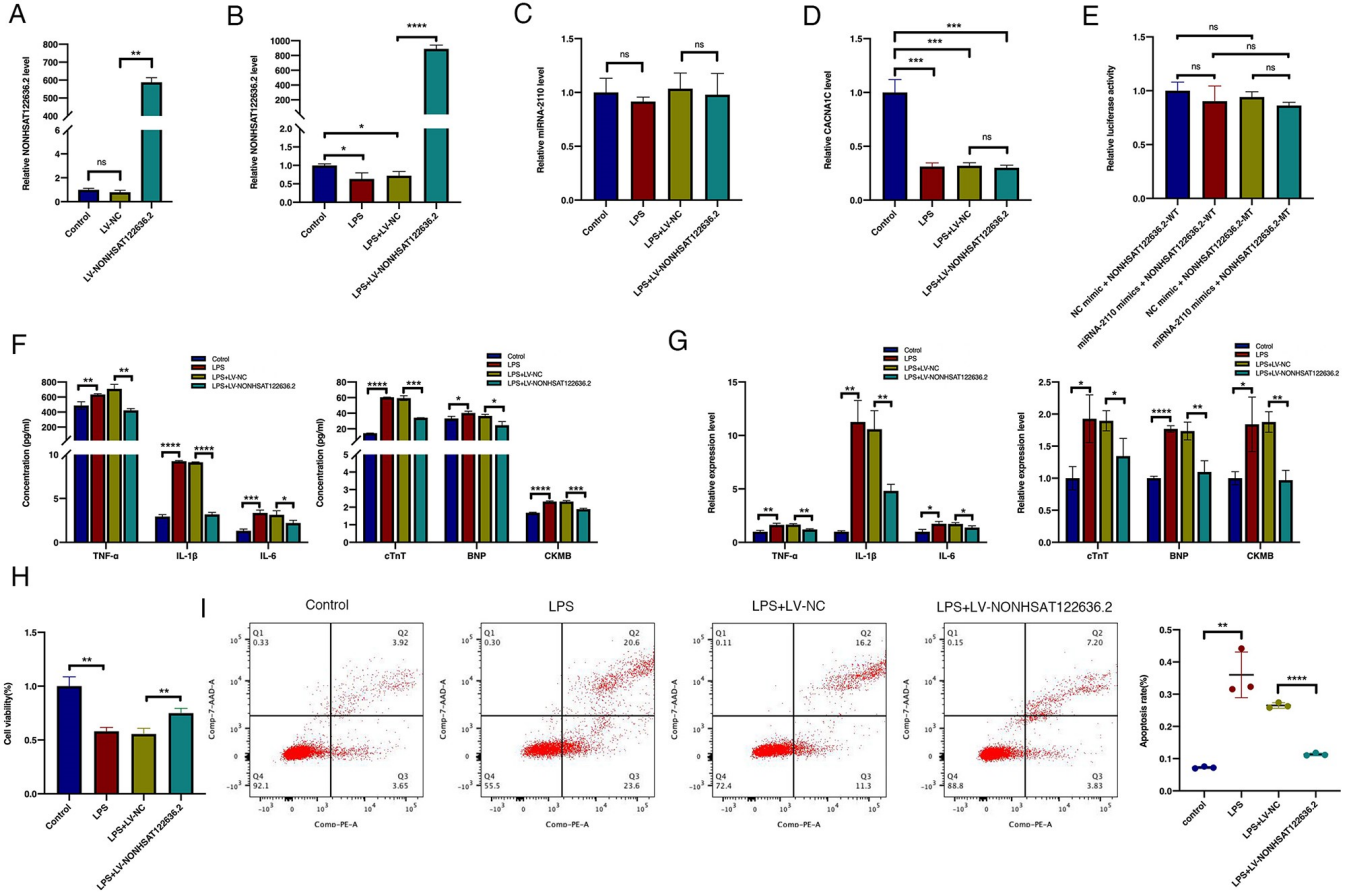
Effect of NONHSAT122636.2 overexpression on inflammatory phenotypes of HCMs. (**A**) LV-NONHSAT122636.2 or negative control (LV-NC) lentiviral vector was transduced in HCMs. NONHSAT122636.2 was significantly overexpressed. (**B-D**) NONHSAT122636.2, miR-2110, and CACNA1C were expressed in transfected HCMs after exposure to 100 μg/mL of LPS for 24 h. (**E**) The dual-luciferase reporter assay indicated a very weak binding force between NONHSAT122636.2 and miR-2110. (**F, G**) The expression levels of IL-6, CKMB, TNF-α, BNP, IL-1β, and TnT were detected by ELISA and qRT-PCR. (**H, I**) Cell viability and apoptosis were measured. Data were presented as mean ±SD. *p< 0.05 versus controls; **p< 0.01 versus controls; ***p< 0.001 versus controls; ****p< 0.0001 versus controls; ns (no significance), *p*> 0.05 versus controls (Student’s t-test).

To further investigate the effect of NONHSAT122636.2 on inflammatory and apoptotic responses in LPS-treated HCMs, we infected HCMs with LV-NONHSAT122636.2 or LV-NC, followed by simulation with 100 μg/mL of LPS for 24 h. NONHSAT122636.2 expression was increased approximately 600 folds in the LV-NONHSAT122636.2 group compared to the LV-NC group (Fig 5A), indicating successful overexpression of NONHSAT122636.2 in HCMs. LPS stimulation did not affect the overexpression of the lncRNA (Fig 5B). Furthermore, overexpression of NONHSAT122636.2 significantly decreased the concentration and expression levels of inflammatory cytokines and myocardial injury markers (Fig 5F and 5G), increased cell viability (Fig 5H), and decreased apoptosis (Fig 5I), these results suggest that activation of NONHSAT122636.2 may have beneficial effects in alleviating inflammatory symptoms related to myocardial injury.

### NONHSAT122636.2 functions independently of the NONHSAT122636.2-miR-2110-CACNA1C axis

According to the ceRNA mechanism, NONHSAT122636.2 and CACNA1C are downregulated, while miR-2110 is upregulated in patients with myocarditis. However, we found that miR-2110 expression was decreased in children with myocarditis (Fig 1F). To determine whether NONHSAT122636.2 functions through the NONHSAT122636.2-miR-2110-CACNA1C axis, we examined miR-2110 and CACNA1C expressions in the four groups (Control, LPS, LPS+LV-NC, and LPS+LV-NONHSAT122636.2) by RT-qPCR. The results showed that miR-2110 expression did not change significantly in both LPS treatment groups with or without LV-NONHSAT122636.2 (Fig 5C). Thus, neither LPS stimulation nor NONHSAT122636.2 overexpression could change alter miR-2110 expression in HCMs. However, CACNA1C expression was significantly reduced in LPS-stimulated HCMs (Fig 5D), and NONHSAT122636.2 overexpression could not activate CACNA1C expression in these cells, suggesting that inflammation can inhibit CACNA1C expression independently of NONHSAT122636.2 overexpression. The dual-luciferase reporter assay revealed a very weak binding force between NONHSAT122636.2 and miR-2110 (Fig 5E). In conclusion, NONHSAT122636.2 may function independently of the NONHSAT122636.2-miR-2110-CACNA1C axis and may involve other signaling pathways that need further investigation.

## Discussion

Myocarditis is a common cardiovascular disease primarily caused by microbial infections and associated autoimmune injuries. Currently, there is no specific diagnostic method for pediatric myocarditis, and clinical diagnosis primarily depends on clinical manifestations, markers of myocardial injury, electrocardiogram (ECG), echocardiogram (ECHO), and cardiac MRI examinations. The specific pathogenesis of myocarditis remains unclear.

Recent studies have highlighted the pathogenic roles of lncRNAs in the initiation and progression of inflammation and autoimmune injuries. The regulation mediated by LncRNA is diverse and complex, influencing gene expression at transcriptional and translational levels depending on their subcellular localization [14]. In the cytoplasm, lncRNAs act as ceRNAs, sequestering miRNAs and thereby derepressing target mRNAs [23]. Zhou Z et al. [24] demonstrated that the lncRNA SNHG16 controls the miR-146a-5p/CCL5 interaction to modulate the LPS-induced apoptosis in WI-38 cells and inflammation in acute pneumonia. Zhuang Y et al. reported that lncRNA-H19 induced senescence in cardiomyocytes by targeting the miR-19a-socs1-p53 axis [25].

In our previous microarray analysis of lncRNA expression profiles in blood samples from patients with myocarditis compared to healthy controls, we identified 3,101 differentially regulated lncRNAs, including 1,645 upregulated and 1,456 downregulated. These lncRNAs were selected based on their involvement in signaling pathways related to inflammation and immunity. Among them, NONHSAT122636.2 emerged as a target lncRNA. We obtained its sequence information from PubMed and its ORF using CPAT, confirming NONHSAT122636.2 as an ncRNA. Additionally, RNA-FISH assay revealed that NONHSAT122636.2 predominantly localized in the cytoplasm, suggesting its potential role in the NONHSAT122636.2-miR-2110-CACNA1C axis.

We demonstrated that the relative expression of NONHSAT122636.2 in the peripheral blood was significantly downregulated in patients with myocarditis, with the AUC value of 0.8857, suggesting that NONHSAT122636.2 might play crucial roles in the pathophysiology of myocarditis. Reddy MA et al. [26] showed that the lncRNA DRAIR is downregulated in type 2 diabetes mellitus and exerts inhibitory effects on the inflammatory phenotype of monocytes/macrophages through epigenetic regulation. He Z et al. [27] reported that the lncRNA Chaer expression is significantly reduced in acute myocardial infarction, thus losing its anti-apoptotic effect on the myocardium. In the present study, we conducted a correlation analysis showing that NONHSAT122636.2 was negatively correlated with myocarditis severity. These results indicate that NONHSAT122636.2 can predict the severity of myocarditis and can be used to evaluate myocarditis prognosis.

Additionally, the lncRNASNP v3 database was accessed to retrieve information on three lncRNAs: NONHSAT254241.1, NONHSAT242632.1, and NONHSAT122636.2. The chromosomal position of NONHSAT254241.1 is chr7:67243631–67244512, with a transcript size of 881 bp. The NONHSAT242632.1 lncRNA has a transcript size of 793 bp and is located at position chr2:151765931–151766724. The NONHSAT122636.2 (LNCipedia transcript ID: lnc-RINT1-4:1) is located at position chr7:105932811–105934611, with a transcript size of 1800 bp. To further validate the function of NONHSAT122636.2, we used the LPS-stimulated HCMs to establish a cardiomyocyte inflammation model. After LPS stimulation, myocardial injury markers and inflammatory factors secreted by HCMs significantly increased, along with enhanced apoptosis and decreased cell viability. these findings indicate that LPS effectively simulated cardiomyocyte inflammation and was suitable for our experimental model. As the LPS concentration increased or stimulation time prolonged, inflammation and apoptosis in HCMs also increased, while the relative expression level of NONHSAT122636.2 consistently decreased. This observation suggests a negative correlation between NONHSAT122636.2 expression and the severity of cardiomyocyte inflammation *in vitro*. When the LPS concentration reached 100 μg/mL and the stimulation time was 24 h, NONHSAT122636.2 expression was significantly reduced, which was used as the optimal stimulation condition for the latter experiments.

Then, to explore the biological effects of NONHSAT122636.2 modulation, we conducted a gain-of-function experiment. We observed that overexpression of NONHSAT122636.2 in LPS-stimulated HCMs led to significant reductions in myocardial injury markers and inflammatory factors accompanied by a remarkable increase in cell viability and a substantial decrease in apoptosis. These findings suggest that NONHSAT122636.2 might function as an anti-inflammatory factor and represent a new potential target for treating myocarditis.

After defining the function of NONHSAT122636.2, we further investigated its specific regulatory mechanism. In our previous microarray analysis, NONHSAT122636.2 was predicted to function through the NONHSAT122636.2-miR-2110-CACNA1C axis. Using clinical samples, we validated the relative expression levels of miR-2110 and CACNA1C, which showed that the latter was consistent with the predicted results, while the former was inconsistent. In an inflammation model using LPS-induced HCMs, CACNA1C expression was significantly inhibited, whereas miR-2110 did not change significantly. Additionally, Additionally, overexpression of NONHSAT122636.2 did not significantly alter the expression levels of miR-2110 and CACNA1C. Furthermore, a dual-luciferase reporter assay revealed a very weak binding force between tNONHSAT122636.2 and miR-2110, strongly suggesting that NONHSAT122636.2 might not function through this predicted axis.

This study has several limitations. First, as NONHSAT122636.2 was significantly downregulated in patients with myocarditis, we did not perform loss-of-function experiments. Second, while we found that NONHSAT122636.2 might be used as a diagnostic marker for myocarditis, this needs validation by robust clinical data with larger sample sizes. Third, the diagnosis of myocarditis in patients was based solely on clinical data, lacking confirmation through pathological myocardial biopsy. Moreover, due to the low sequence conservation of lncRNAs, no animal experiments were conducted in this study. Future studies will utilize appropriate animal models to further investigate the relationship between NONHSAT122636.2 expression and the severity of myocarditis at the pathological level.

## Conclusion

NONHSAT122636.2 expression is significantly decreased in patients with myocarditis and is negatively correlated with myocarditis severity. NONHSAT122636.2 might be used as a diagnostic biomarker for pediatric myocarditis. It attenuates myocardial inflammation and apoptosis in patients with myocarditis.

## Supporting information

S1 File (ZIP)

S2 File (ZIP)

S3 File (ZIP)

S4 File (ZIP)

S5 File (ZIP)

S6 File (ZIP)

S7 File (ZIP)
