# Correction: Long noncoding RNA NONHSAT122636.2 attenuates myocardial inflammation and apoptosis in myocarditis

**DOI:** 10.1371/journal.pone.0321699

**Published:** 2025-04-02

**Authors:** Yongjiao Liu, Li Zhang, Hailin Jia, Xinxin Feng, Mengjie Ma, Jing Wang, Bo Han

There is an error in Fig 2. The labels in Fig 2C are incorrect. Please see the correct Fig 2 here.

**Fig 2:**
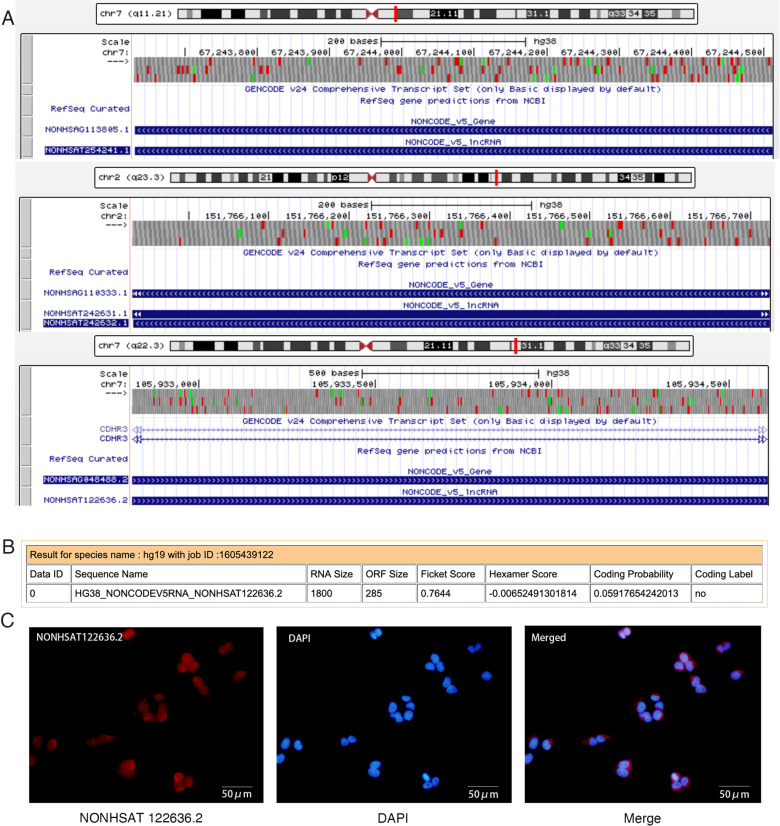
Characterization of the 3 lncRNAs. (**A**) Locations of NONHSAT254241.1, NONHSAT242632.1 and NONHSAT122636.2 on human chromosomes. (**B**) NONHSAT122636.2 was predicted to be a non-coding RNA by the CPAT program. (**C**) FISH assay revealed the predominant localization of NONHSAT122636.2 in the cytoplasm. NONHSAT122636.2 probes were labeled with Cy3 (red). Nuclei were stained with DAPI (blue). Scale bars, 50 μm. CPAT, the Coding-Potential Assessment Tool; FISH, fluorescence in situ hybridization; Cy3, cyanine 3.
