# Supplementary material for: Long noncoding RNA NONHSAT122636.2 attenuates myocardial inflammation and apoptosis in myocarditis
Source: PLoS One. 2024 Aug 16;19(8):e0307779. doi: 10.1371/journal.pone.0307779 (PMC11329147; doi:10.1371/journal.pone.0307779)

**Information about 3 lncRNAs（http://www.noncode.org/）**

>NONHSAT254241.1

gagttcactgaggcccccactctgccgttttggaaCTGATCTCATTTCATTTTAATTATTTAGGTAGCATATTTTAATATAAACCAACTTTCACTTAATAGTTATTTTTTCTAAAATTTCTTAACTATTGTCTGTTTGGCTAATTTTTCAGTTGAATGTTAGAATCATTTTCTTAAGCTCCAAAAAGAAATTCTGATAGAACTTGGGATGTTATTTTGCCAACAGATCACCTTCTTAGAACATTCAGGTTTCCCATTCTCTCCCACTTTTATACTGTTCAGCAAACTTAATGAATGTTTTCATGTAGATTGCATTGTATTTTCCTTAGACATTTCAAATGTTCTGTTGTGACTGTGAACTGGGCTTAAAGTGCTGTTATTTTAAATATGAATCAATACATCAATATTTTCTGGGATGAAAGAAAAACACTCATCTATCAATGAGAATCAGGAGATTGAgatcacgaggtcaggagattgagaccatcctggataacacagtgaaaccccgtctctactaaaaatacaaaaaattagccaggcgtggtggcgggtgcctgtggtcccagctactggggaggctgaggcaggacaatgacgtgaacctgggagacggagcttgcagtgagctgagattgtgccattgcactccagcctgggcgacagagcgagactctgtctcaaaaaaaaaaaaaaGTTAATGGAATCAGGAGGGTTCATTCTGTAGGTAAGAGGTTTGCTTTTTTTTTTTCTTTGAAACAATAAAATATCTTTGTTCAATTTAAATCTTGAGGCCACTCATggtggctcacacttataatcacagcactttgggaggctgaggtgggcggatcacttgaggtcaggagtttgagactagcc


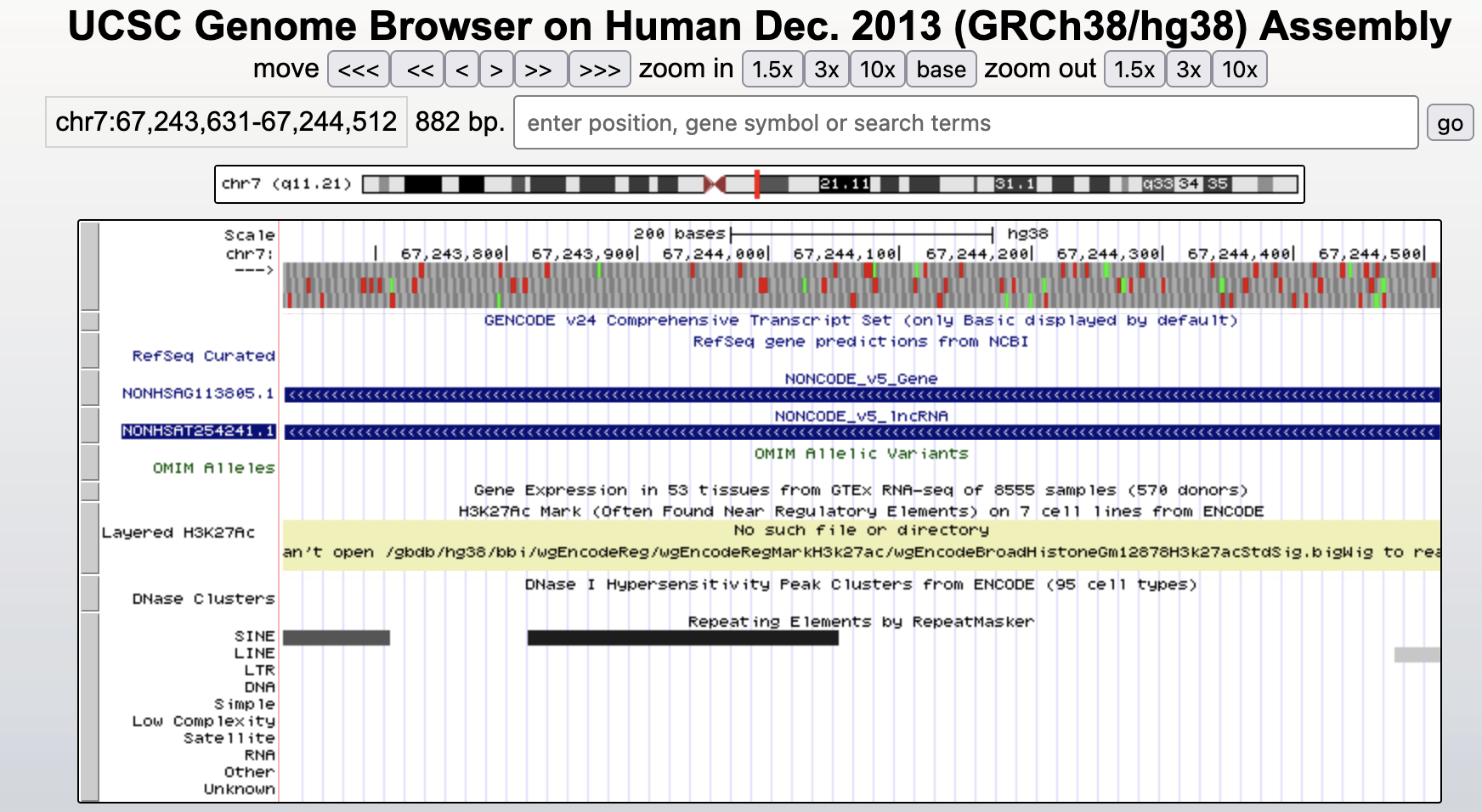


>NONHSAT242632.1

AGCAACGCAGCTTGTTGGTGGAGCTACTGAGCAAAGCCTCTTACCTTCTCTGCTTCTTCAAGGCCCAGTGCTTTATCTGTCAAATTTGGAGAAGATCTCTGCCTTGTAGGCTTTAAATGTTTACTTAGTAGCTCTCAACTTCCTTTCTCTCTTGGCTCAAATTTTGAATGTTGGGGCAGGAAACTACTACTGAATATCATTTACTTGAGATTTCCCTTGTGGCCCATTAGAAGGAgtgtgtgtgtgtgtgtgtgtgtgCATGAGAGAGACACAGAGAGAACACACCAAAGGCCACTGTGGGTgagggtggggtgtttggatgaacaccagtctggaagaatcaggacccctggacttctaggcctgaccccactgctccctggtcatgtggctttggggaagccactctaacctttctaggcctcattttctccatgtctaaaatgaataccataaATAAGAAGTAATTTCTAGTGTATTATGCTACTCAAACTTCTGTTCTCATTTGTTTTTGTGTGTACCTTCTGCATGCCAGGAACTAATTCACAGCATGAAACAAGCCCCTGCAGTCCCAACTCTTAGCAGCCCGTTCCCCCTGGGAGAGGTCAGGTCACACCCAGATAGACAGCTCTACAACGGAAGGTAGAGGTGATGAGCAGCATGCAGGCAATACGGCAATACAGCGAGGCAGGGGCGTGGAGAGCGATGGGCAGGCGCTGTTTGAGCAGGGAGCTTAAGCACTTGAGGCAGTGAGTCCAGAGTGTGGCCTGAGCACGGAGGGGCAGGAAGTGGG


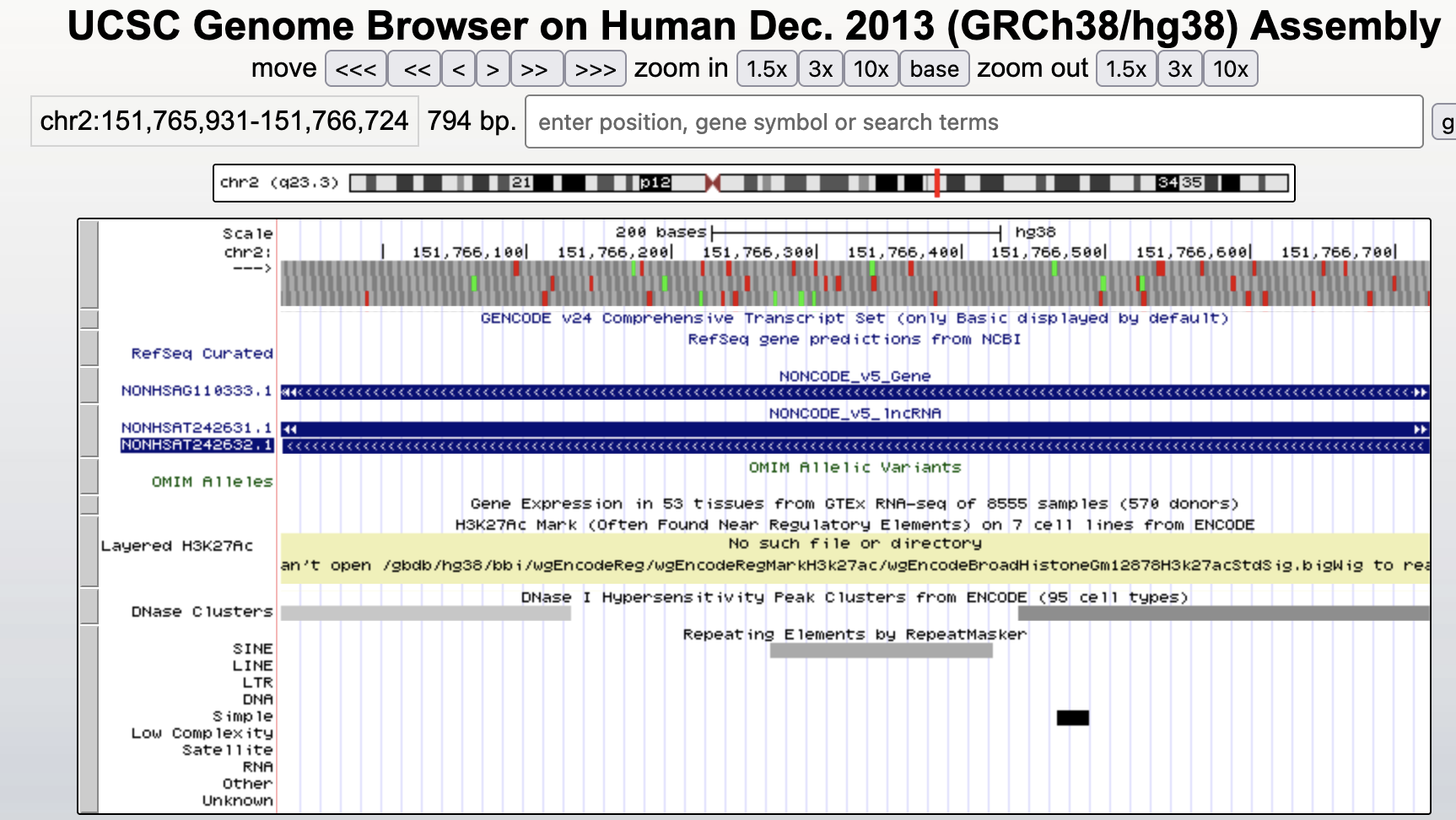


>NONHSAT122636.2

CTATTGCTCCCCTTCTCTCTTTGTACTCAGAAAATCCAGGAAATTCTTCCTCAAGAAAGCATGACCCATTTCAGCCTAAAAGCGTGCTTTTCTCTGGACTGTGCTGGCTTGAGAACATATTTCTTTTACCAAAAAatgagttcacagtaagtgactcagaagcatatggatgaaatttcctttaagccactcaagggattagaaattaagggaaagagtgcctggcaaaatgcatttggggttttgccaagcccaaaccaactgagaaatagttaagacacaaggcagatctcaaatgctgccaagtttacccatcTTAGCAGGACAGGGATTTATAATTAGAGCTGCTCAGAAAAGCACTGCCTTACGGGTGGCAAAGGACAAAAAAGGCTTTGTCCCCAACATCTCCACAGGCTGCGGTTTCCCAGATGGGCCCTTAACAGTGTACACACCCTCTCCATCGCCAGGGTCCTCAAAGCCACCGCCTCTACCAAGCAAGGTTCGACTTCTACACCACGCCCCTGACATTCTCTCCAGGCCTCTTTTGTTTCTGGCCCAGGCTGGATGCATCACACTGTGAATTCCTGGATGAGGAAGAACAAATCTTGCGGAGTGTTCTGGAATTACAGAGGAGAGCCATAATGGGAAAATGTGACAAAGCAATTCCCTTCTCTGACATACACATTTTACCCCTGTCTCTCACCTTTGTCACGTTTCCCCATTTTttcaccttctgtgggtttctttttctgtaaaatgaagggattggatctgctggtttcaaaCTGTGCTACCTGGAGCCACTTGCGGGCCTGGCGGGGCCCAGTGGAAGTGGATGGGGAGATGGTGAGAAGGGCTCTCTTCAAAAAAAGCAGGAGGGGGCTCCAACATAGCATTTCAAGGAAATCAAGGGATCCAAAGTTAAACAATTTGAAAATCACAAGTCTGGACGAAGTCTCATTGTTCTTTCCAACTGAACACTGCCGGTGTGGTGTGCCTCACTTCTCAGTCTCCATTCACTAACCCAAATATCCCCAAAGATGTGCACCAATTTTTCTGTCTAGCATTCATCAGAGTGctcctgtgtgccaggccctctgctaagcactgggcatacagcagtgaacagaaaccccagggggctcatagaccagttgggaagattggcattaaacaaatgaatatgtaaatccaaaTCAAGAGGTATGAACACCATGAAAGACAAAATCATGGTTGAAAGTTGAGTGGGCCTGGGGTTGGAGAGATACTGCCCTAAGCATCTCCCCTCCCCACCTGGCTTGCCACTTGCCCCTTTATAGAATAACTAATCGTTGTTCAGTCTTTGATAGTTTCCACTGAAAACATCCCTCTTCTACCTGACAGAGGAGGGTCAAGACACCCAGAGCACAAAGGAGCTGATGGGTTTAGTTAGCTGTTACTGCGGAAAGCCAAGAGGATGAGCTTGAGAGAACAGACTGAGGGCTTTAGATGGGTGAATGTGAGAAAGTCTACAGTGCTTGCTGTGCTGAGGAAAAAATCCAGGTGGATCACCTGGAGGTGACCCAAAGTGCCAATGAAGTGCATGTCAATGGCCCTCTTCACATACTTCACTACTGTATCTGGAGCTCTCCTGAACATCTTTCTCCCATTCCTCACTGAGTTTTGCTAGACATGGCAAATGTTAACTCATTACCAACTCTTAATTGTATTGCTGTTTCTTTGCCCCTGTCTAGCTCTTTCTATTTGTACATTTTGTAAAGAGAAAAGGAATTTAAAAAATAAAACCAAAACAATCTCTGG


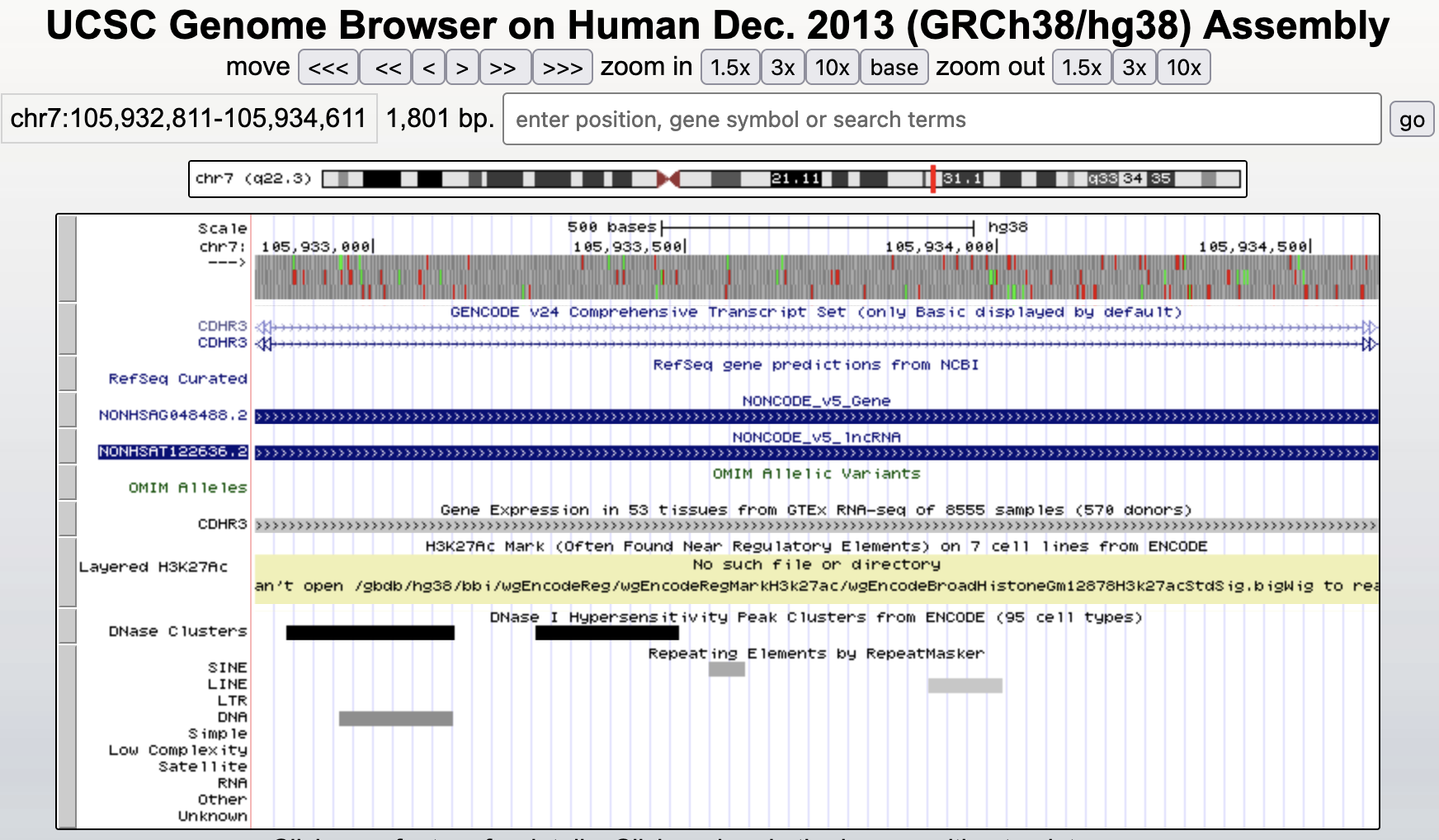

Supplement: S2 File — (ZIP) [file pone.0307779.s002.zip › Information about 3 lncRNAs.docx]
