# Supplementary figures and images for: Long noncoding RNA NONHSAT122636.2 attenuates myocardial inflammation and apoptosis in myocarditis
Source: PLoS One. 2024 Aug 16;19(8):e0307779. doi: 10.1371/journal.pone.0307779 (PMC11329147; doi:10.1371/journal.pone.0307779)

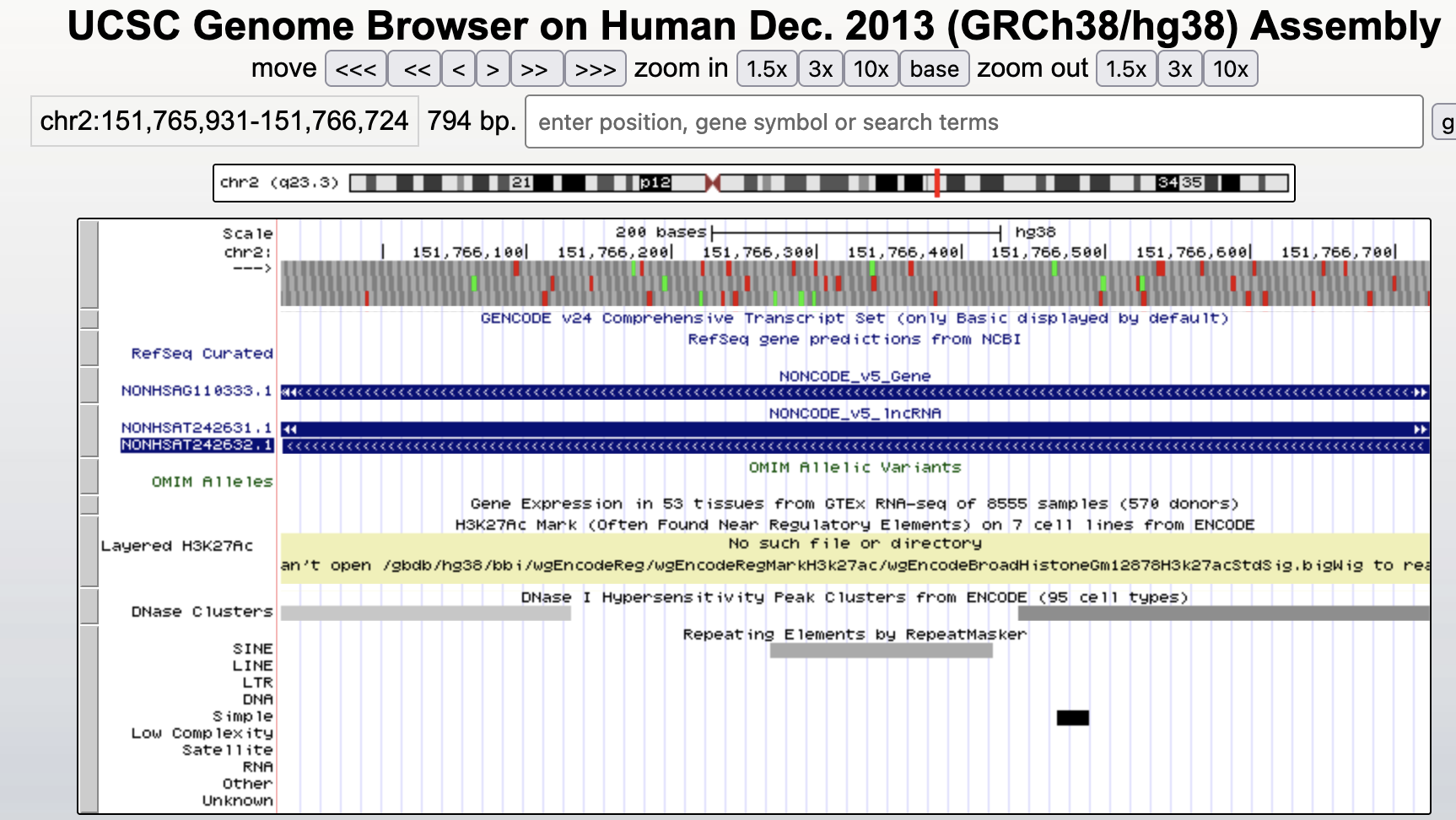

Supplement: S2 File — (ZIP) [file pone.0307779.s002.zip › 2023-12-30 9.42.50.png]

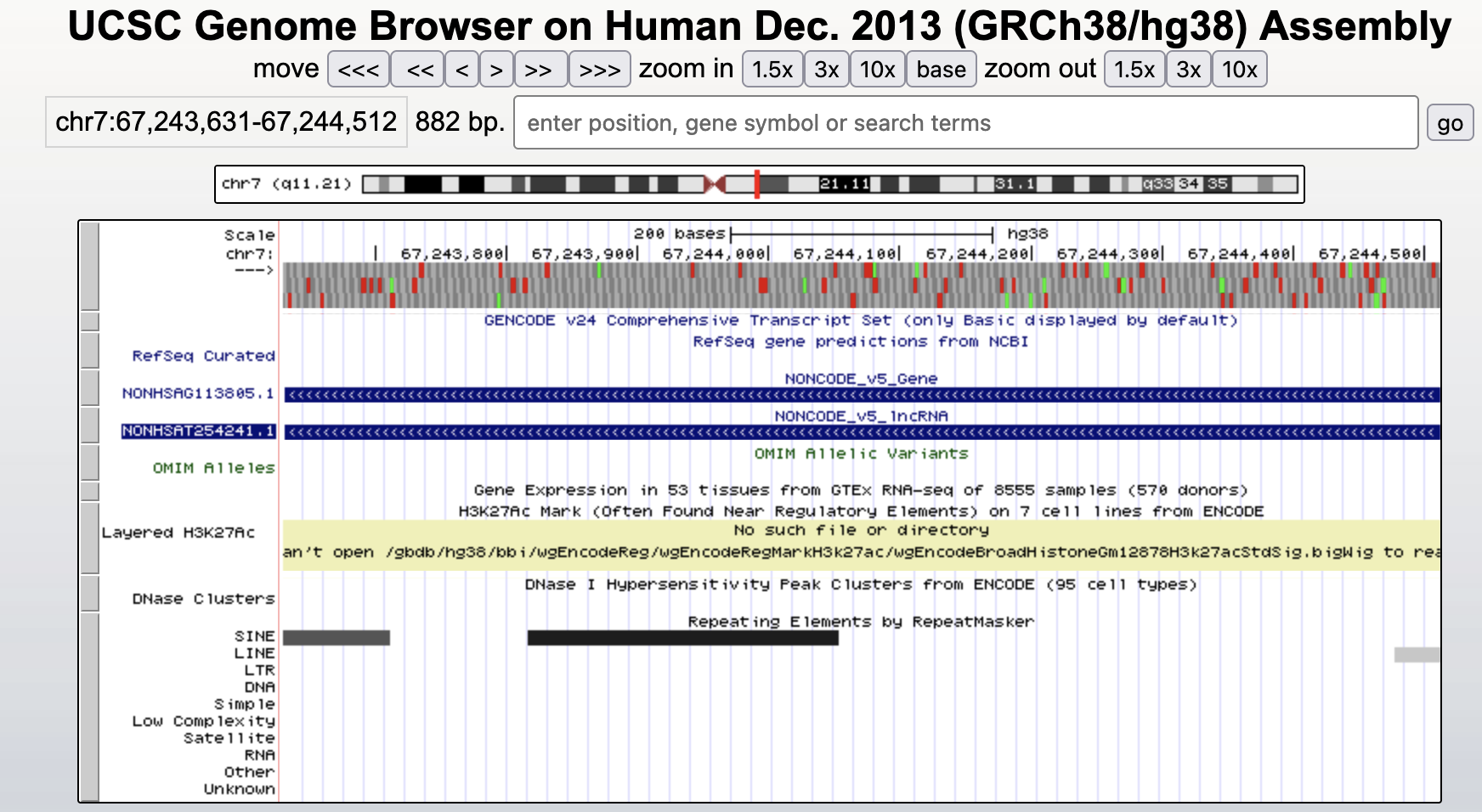

Supplement: S2 File — (ZIP) [file pone.0307779.s002.zip › 2023-12-30 9.46.18.png]

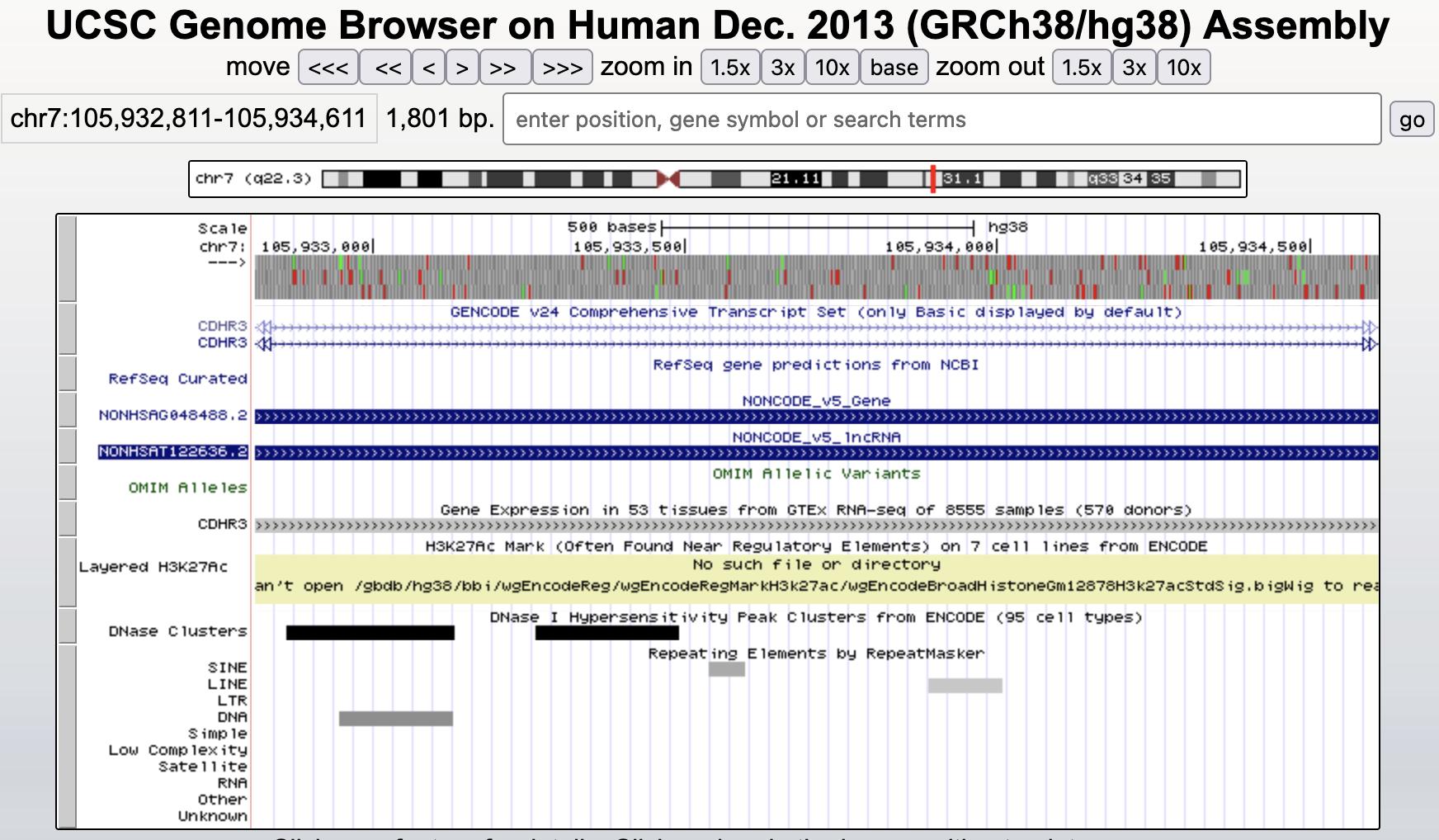

Supplement: S2 File — (ZIP) [file pone.0307779.s002.zip › 2023-12-30 9.47.40.png]

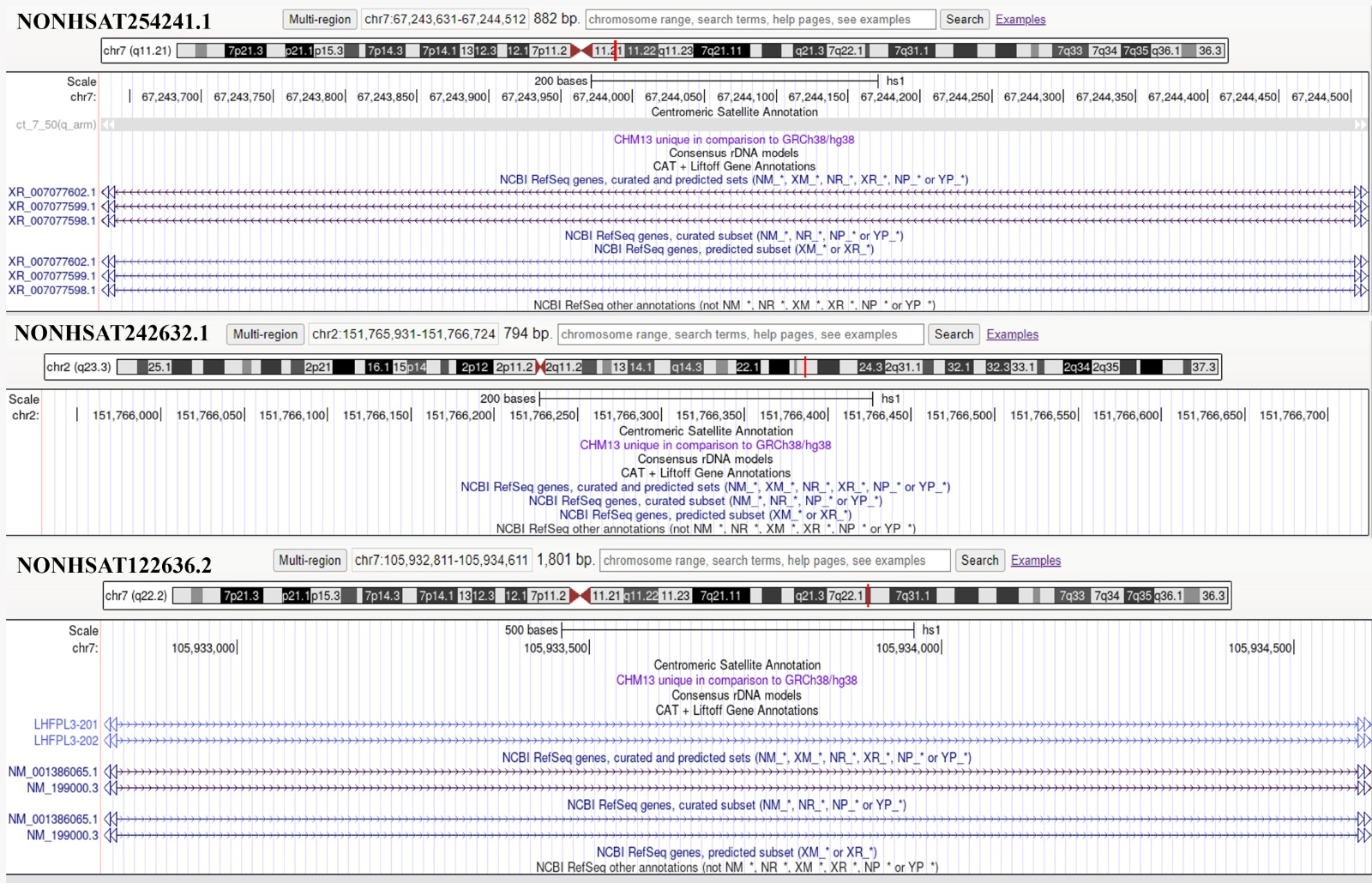

Supplement: S2 File — (ZIP) [file pone.0307779.s002.zip › Genome browser screenshots of the surrounding genes.jpg]

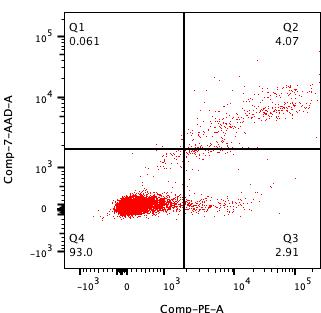

Supplement: S6 File — (ZIP) [file pone.0307779.s006.zip › 0μg_ml 24h ( 100μg_ml 0h) (control)/Figure4D.jpg]

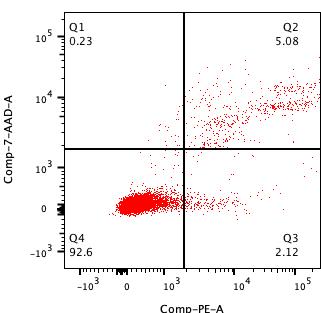

Supplement: S6 File — (ZIP) [file pone.0307779.s006.zip › 0μg_ml 24h ( 100μg_ml 0h) (control)/Figure4H-1.jpg]

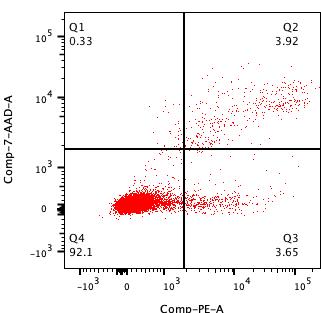

Supplement: S6 File — (ZIP) [file pone.0307779.s006.zip › 0μg_ml 24h ( 100μg_ml 0h) (control)/Figure5I-1.jpg]

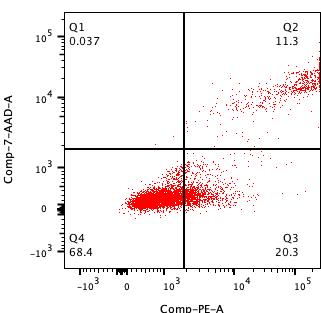

Supplement: S6 File — (ZIP) [file pone.0307779.s006.zip › 100μg_ml 24h (LPS)/Figure4D-3.jpg]

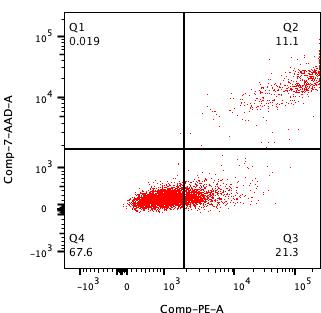

Supplement: S6 File — (ZIP) [file pone.0307779.s006.zip › 100μg_ml 24h (LPS)/Figure4H-3.jpg]

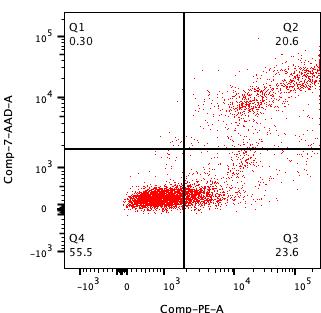

Supplement: S6 File — (ZIP) [file pone.0307779.s006.zip › 100μg_ml 24h (LPS)/Figure5I-2.jpg]

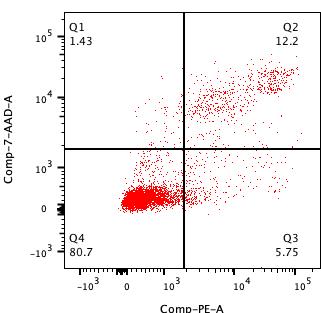

Supplement: S6 File — (ZIP) [file pone.0307779.s006.zip › 100μg_ml 6h/10.jpg]

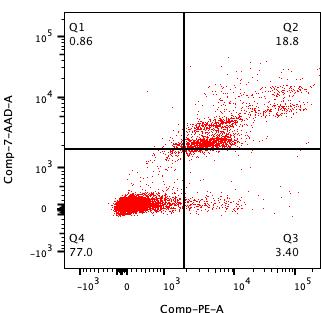

Supplement: S6 File — (ZIP) [file pone.0307779.s006.zip › 100μg_ml 6h/11.jpg]

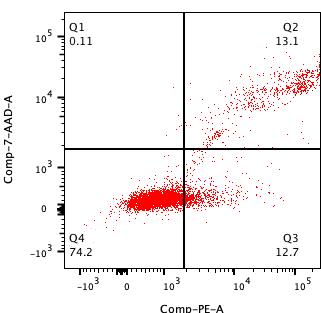

Supplement: S6 File — (ZIP) [file pone.0307779.s006.zip › 100μg_ml 6h/Figure4H-2.jpg]

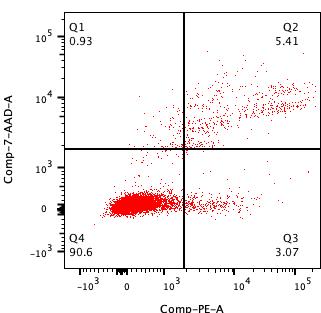

Supplement: S6 File — (ZIP) [file pone.0307779.s006.zip › 10μg_ml 24h/4.jpg]

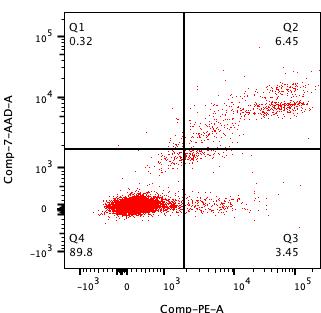

Supplement: S6 File — (ZIP) [file pone.0307779.s006.zip › 10μg_ml 24h/6.jpg]

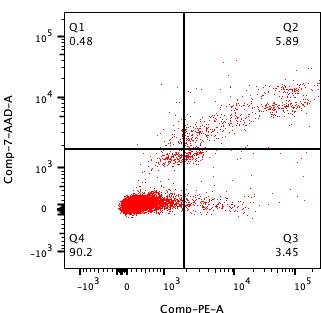

Supplement: S6 File — (ZIP) [file pone.0307779.s006.zip › 10μg_ml 24h/Figure4D-2.jpg]

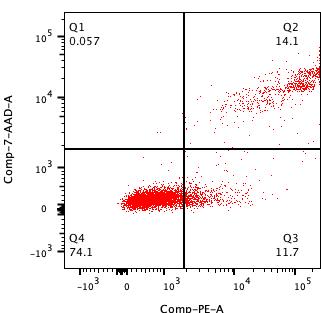

Supplement: S6 File — (ZIP) [file pone.0307779.s006.zip › LPS+LV-NC/13.jpg]

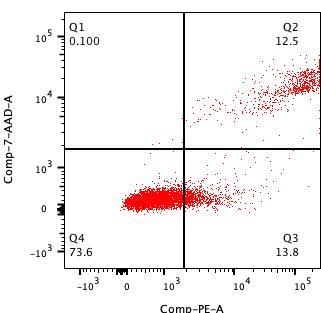

Supplement: S6 File — (ZIP) [file pone.0307779.s006.zip › LPS+LV-NC/14.jpg]

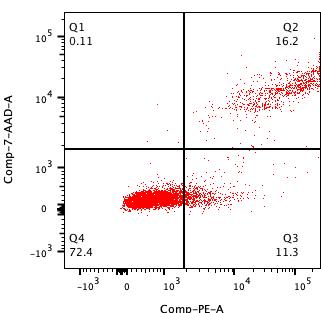

Supplement: S6 File — (ZIP) [file pone.0307779.s006.zip › LPS+LV-NC/Figure5I-3.jpg]

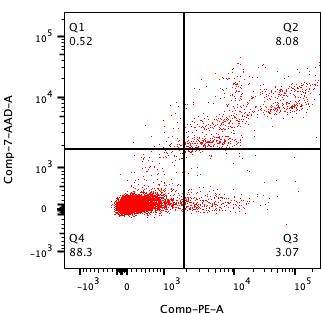

Supplement: S6 File — (ZIP) [file pone.0307779.s006.zip › LPS+LV-NONHSAT122636.2/17.jpg]

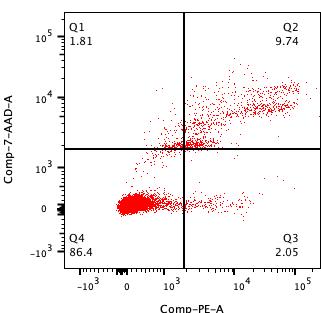

Supplement: S6 File — (ZIP) [file pone.0307779.s006.zip › LPS+LV-NONHSAT122636.2/18.jpg]

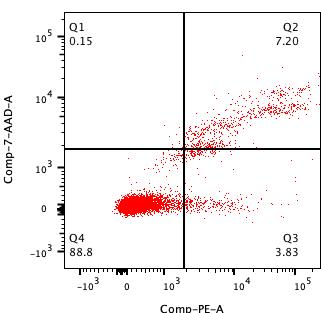

Supplement: S6 File — (ZIP) [file pone.0307779.s006.zip › LPS+LV-NONHSAT122636.2/Figure5I-4.jpg]
